# Circular RNA Plek promotes fibrogenic activation by regulating the miR-135b-5p/TGF-βR1 axis after spinal cord injury

**DOI:** 10.18632/aging.203002

**Published:** 2021-05-11

**Authors:** Wenzhao Wang, Dong He, Jianan Chen, Zhengdong Zhang, Shaoyi Wang, Yunpeng Jiang, Jianlu Wei

**Affiliations:** 1 Orthopedic Research Institute, Department of Orthopedics, West China Hospital，Sichuan University, Chengdu, Sichuan, China; 2 Department of Orthopedics, Qilu Hospital, Cheeloo College of Medicine, Shandong University, Jinan, Shandong, China; 3 Department of Neurology, Cheeloo College of Medicine, Shandong University, Jinan, Shandong, China; 4 Department of Orthopedics, Cheeloo College of Medicine, Jinan Central Hospital Affiliated to Shandong University, Jinan, Shandong, China; 5 Department of Orthopedics, The First Affiliated Hospital of Chengdu Medical College, Chengdu, Sichuan, China

**Keywords:** spinal cord injury, circular RNA, fibroblast, miR-135b-5p

## Abstract

Objectives: The spinal cord rarely repairs itself when damaged; however, methods for encouraging nerves to regrow are on the horizon. Although circular RNAs (circRNAs) contribute to various biological processes, including neuronal processes, their functions in the subacute phase of spinal cord injury (SCI) have not been elucidated. In this study, we identified a novel circRNA, named CircPlek, with increased expression in spinal tissues after SCI.

Materials and Methods: We predicted a regulatory relationship between CircPlek and miR-135b-5p, which showed the most obvious decrease in post-SCI expression. We established the CircPlek/miR-135b-5p/transforming growth factor-beta receptor type I (TGF-βR1) axis using a bioinformatics approach and further evaluated the potential function of the interaction network *in vitro*.

Results: We confirmed that in TGF-β1-induced fibroblasts, the overexpression of miR-135b-5p or/and inhibition of CircPlek inhibited fibrosis activation via the Smad pathway. Inhibitors of miR-135b-5p had antagonistic effects on CircPlek.

Conclusions: the CircPlek/miR-135b-5p/TGF-βR1 axis may exert important functions in SCI and is a potential therapeutic target.

## INTRODUCTION

Spinal cord injury (SCI) is a disabling and irreversible central nervous system injury with high economic and social costs [1–3]. It has a number of underlying causes, including trauma, vascular lesions, infection, and tumors [4]. The most common cause is traffic accidents; however, with the aging of society, falls account for an increasing number of cases each year [3]. Peripheral nerve fibers that relay neural signals and innervate muscles project from the spinal cord. Unlike the peripheral nerve, the spinal cord rarely repairs itself when damaged [5]. For 4,500 years, doctors have struggled to improve recovery from SCI, and practical, meaningful, and clinically translatable treatments have yet to be developed [1]. More than half of the patients lose total motor and sensory function in areas controlled by nerves that exit below the site of damage [3]. SCI also causes a wide range of physical and mental disorders, such as sleep disorders, depression, sexual dysfunction, and obesity [6]. Axon shearing and Wallerian degeneration begin with physical trauma, followed by blood–spinal cord-barrier damage, ischemia, cell necrosis, and apoptosis, known as the primary injury phase [7]. However, severe damage occurs during secondary injury, in which inflammation is evident and, most strikingly, barriers that prevent axon regeneration form [4]. Although repair and regeneration occur in response to damage, scar tissue formation makes these processes even more difficult. Fibrous scars, composed of fibroblasts, and glial scars, composed of astrocytes, form in the subacute stage of SCI. These scars not only physically block axonal regeneration but also secrete a large number of chemical inhibitors [8–10]. Effective treatment regimens require a more detailed understanding of the underlying mechanisms. Despite vast improvements in our understanding of the extrinsic and intrinsic factors that block axonal regeneration or neuroplasticity, it is still not enough to find solutions.

Non-coding ribonucleic acids (ncRNAs) are a class of genetic, epigenetic, and translational regulators with essential roles in various physiological and pathological processes [11]. At least 70% of the human genome is transcribed, but protein-coding genes only account for approximately 2% of the transcribed portion [12]. Accumulating evidence indicates that ncRNAs, especially microRNAs (miRNAs) and circular RNAs (circRNAs), are involved in nervous system disorders and the associated pathophysiology [13, 14]. CircRNAs, a novel type of endogenous ncRNA, are abundant yet enigmatic in mammalian cells and form by back splicing resulting in covalently closed continuous loops, without open reading frames, 5′ caps, or poly-A tails [15, 16]. There are more circRNAs in nervous system tissues than in other tissues, and neuron-specific genes often have long introns and carry more sequence features that promote circRNA formation during development, synapse formation, neuronal differentiation, and neuronal stimulation and plasticity [15]. Although their physiological functions are not yet completely defined, one of the most well-studied functions of circRNAs is their role as miRNA sponges. For instance, CiRS-7 acts as an miR-7 sponge, inhibiting the post-transcriptional regulation of miR-7 in central nervous system (CNS) neurons [17]. Comprehensive analyses of circRNAs in the spinal cord will facilitate the identification of candidate diagnostic biomarkers and therapeutic targets for SCI.

We have previously identified various miRNAs that regulate scar formation after SCI. For example, miR-21a-5p acts as a negative regulator of SCI recovery in the acute phase via the TGF-β/Smad and PI3K/Akt/mTOR signaling pathways [16, 18–20]. In this study, we predicted and analyzed differentially expressed circRNAs, miRNAs, and mRNAs in the sub-acute stage of a spinal cord lesion epicenter of a modified Allen’s weight drop model of SCI using RNA sequencing and bioinformatic approaches. After functional enrichment analyses, a circRNA/miRNA/mRNA interaction network was constructed and validated. Our results are expected to provide a better understanding of the diagnostic, prognostic, and therapeutic value of ncRNAs in SCI.

## MATERIALS AND METHODS

### Identification of differentially expressed RNAs and extraction of spinal tissues

Raw data from previous sequencing were reanalyzed in conjunction with the latest tools [21], differentially expressed reads were identified by a bioinformatics approach. Briefly, differential expression based on normalized deep-sequencing counts was analyzed by the Fisher exact test, chi-squared 2 × 2 test, chi-squared n × n test, and Student’s *t*-test. The thresholds for the differential expression of mRNAs and ncRNAs were log2^(fold change)^ >1 or log2^(fold change)^ <−1 and *p* < 0.05. StringTie (http://ccb.jhu.edu/software/stringtie/) was used to evaluate mRNA expression levels, miRNAs were identified using miRBase 22.1 (http://www.mirbase.org/) and CIRCexplorer was used to de novo assemble the mapped reads to circRNAs [22]. All procedures involving animals were approved by the Ethics Committee of Shandong University and were performed in compliance with the National Institutes of Health Guide for the Care and Use of Laboratory Animals. Thirty clean, healthy, 8-week-old male C57BL/6 littermates were purchased from the Laboratory Animal Center of Shandong University. Spinal tissue samples were obtained as previously described [21]. In brief, mice were randomly divided into SCI and Sham groups. Mice in the SCI group were subjected to the modified Allen’s weight drop injury. Mice in the Sham groups only underwent vertebral laminectomy without SCI. Six mice from each group were randomly selected for hematoxylin–eosin (HE) staining at 1, 3, and 7 days post-surgery. The remaining mice were used for RNA and protein extraction at 1, 3, 7 and 14 days post-surgery. HE staining was performed as described previously; briefly, the spinal cord tissues were stripped off, fixed with 4% paraformaldehyde, paraffin-embedded, dewaxed with xylene, placed in an ethanol gradient, and stained with HE [21].

### Splice junction and PCR validation

Total RNA from tissues was isolated using MagZol Reagent (Magen, Guangzhou, China). RNA concentration and quality were determined by ultraviolet spectrophotometry (absorbance at 260 nm/280 nm, Invitrogen, Carlsbad, CA, USA). cDNA was synthesized using the TUREscript 1st Stand cDNA SYNTHESIS Kit (Aidlab Biotechnologies, Beijing, China). RNA (500 ng), 4 μL 5× RT Reaction Mix, 0.8 μL Rondam Primer/oligodT, and 0.8 μL TureScript H-RTase/RI Mix were used for the reverse transcription of mRNA in a reaction system of 20 μL. The reverse transcription reaction conditions were 42°C for 40 min and 65°C for 10 min. The reverse transcription of miRNA was carried out in a reaction system of 10 μL, consisting of 200 ng total RNA2, 2 μL 5× RT Reaction Mix, 0.5 μL RT primer, and 0.5 μL TureScript H-RTase/RI Mix, with RNase-free dH2O added to 10 μL. The reaction conditions were 25°C for 10 min, 42°C for 50 min, and 65°C for 15 min. Real-time qPCR was performed using SYBR Premix Ex Taq (Takara, Dalian, China) according to the manufacturer’s instructions. The primers were as follows: m-circRNAPlek-validation-220 bp-R1 GAACATCCGTTT GCCAAAGTC; m-CircRNAPlek-DP-220 bp-F1 CCACTTCCAAGGAGCGTACTG; m-CircRNAPlek-DP-220 bp-R1 GAACATCCGTTTGCCAAAGTC; mmu-miR-135b-5p-F GGCGGTATGGCTTTTCATTC; mmu-miR-135b -5p-RT GTCGTATCCAGTGC AGGGTCCGAGGTATTCGCACTGGATACGACTCACAT; TGF-βR1 GAAAGCATTGGCAAAGGTC GGTTT; TGF-βR1 CTCTCGGAACCATGAACG CTCTT; U6-F CTCGCTTCGGCAGCACA; U6-R AACGCTTCACGAATTTGCGT; GAPDH-F GTGGT GAAGCAGGCATCT; GAPDH-R GGTGGAAGAGT GGGAGTTG. The expression of *GAPDH* was used as an internal control for relative circRNA and mRNA expression level normalization, whereas U6 was used as the internal control for miRNA expression. The 2^–ΔΔCT^ method was used for comparative quantitation. Data are presented as the means of three experiments. Sanger sequencing was performed in accordance with the manufacturer's instructions (RiboBio, Guangzhou, China). Reverse-transcriptase polymerase chain reactions (RT-PCR) were carried out in accordance with the manufacturer's instructions of RT kit (RiboBio). PCR products were separated by electrophoresis on an agarose gel (1.5%) at 120 V of constant pressure for 30 min.

### Functional, pathway, and network analyses

A GO analysis was used to identify enriched functions of the differentially expressed genes in three categories, cellular components, biological processes, and molecular functions (http://www.geneontology.org) [23]. The KEGG database (http://www.genome.ad.jp/kegg/) was also used to analyze the potential functions of target genes in the pathways [24]. An ncRNA regulatory network was constructed to reveal the interactions in subacute SCI pathological processes by identifying functional links between dysregulated circRNAs, miRNAs, and mRNAs. miRNA target genes were predicted using TargetScan (http://www.targetscan.org/), miRecords and miRBase, as described previously [25]. Cytoscape (San Diego, CA, USA) was used to build the CircRNA/miRNA/mRNA interaction network. The STRING database (http://string-db.org/) was used to analyze the PPI network with a combined score of >0.4 as the cutoff value.

### Luciferase assay and fluorescence

CircRNAPlek with wild-type and mutant binding sites for miR-135b-5p as well as the TGF-βR1 promoter region sequence were validated using a dual-luciferase reporter system (Promega, Madison, WI, USA). Firefly and *Renilla* (Invitrogen) were transfected with 0.25 miRNA transfection reagent (Invitrogen) for 48 h and luciferase activity was measured. The Fluorescent *In Situ* Hybridization Kit (RiboBio) was used according to the manufacturer’s instructions. Fluorescence was detected using an Opera Phenix HCS system (PerkinElmer, Waltham, MA, USA).

### Cell culture and treatment

Primary spinal fibroblasts (PriCells, Wuhan, China) were cultured as described previously [25]. Transfection with siRNA-circRNAPlek (CACGGACTGGGAA GGAAGT), miR-135b-5p mimics, and negative controls (RiboBio, Guangzhou) was initiated when the cells were 30%–50% confluent. After 48 h, the cells were stimulated with TGF-β (10 ng/mL; ACRO, Beijing, China) for 48 h.

### Western blot analysis and immunofluorescence

Protein was harvested from cells and tissues as described previously [20]. Total proteins were obtained via a radio-immunoprecipitation assay (Sigma-Aldrich, St. Louis, MO, USA). Proteins were subjected to sodium dodecyl sulphate polyacrylamide gel electrophoresis (Solarbio, Beijing, China) and transferred onto polyvinylidene fluoride membranes (Millipore, Billerica, MA, USA). The membrane was blocked with 5% skim milk powder and incubated at 4°C overnight with the following primary antibodies: anti-fibronectin antibody (Abcam, Cambridge, UK), type I collagen antibody (Abcam), anti-p-Smad2 antibody (Cell Signaling Technology, Danvers, MA, USA), anti-p-Smad3 antibody (Cell Signaling Technology), anti-Smad2/3 antibody (Cell Signaling Technology), and anti-GAPDH antibody (1:5000; Abcam). After probing with a secondary antibody (Solarbio), the blots were visualized using West Pico enhanced chemiluminescent substrate (Solarbio). Immunofluorescence of fibroblasts was performed as previously described [25]; in brief, after washing, permeating, and blocking, cells were incubated with Type I collagen antibody (Abcam) and Alexa Fluor^®^ 594 goat anti-rabbit IgG secondary antibody (Thermo Fisher Scientific, Waltham, MA, USA). Immunofluorescence was analyzed under a fluorescence microscope (Olympus Corporation, Tokyo, Japan) after staining with 4′,6-diamidino-2-phenylindole (DAPI; Thermo Fisher Scientific).

### Statistical analysis

Statistical analyses were performed using GraphPad Prism (GraphPad Software, La Jolla, CA, USA). Data are presented as means ± SD. ANOVA and Student’s *t*-test were used to assess statistical significance, with a threshold of *p* < 0.05.

### Ethical approval and consent to participate

All experiments were reviewed and approved by the Ethics Committee of Shandong University.

### Data availability statement

The data that support the findings of this study are available from the corresponding author upon reasonable request.

## RESULTS

### Identification of dysregulated ncRNAs and mRNAs

**Figure 1:**
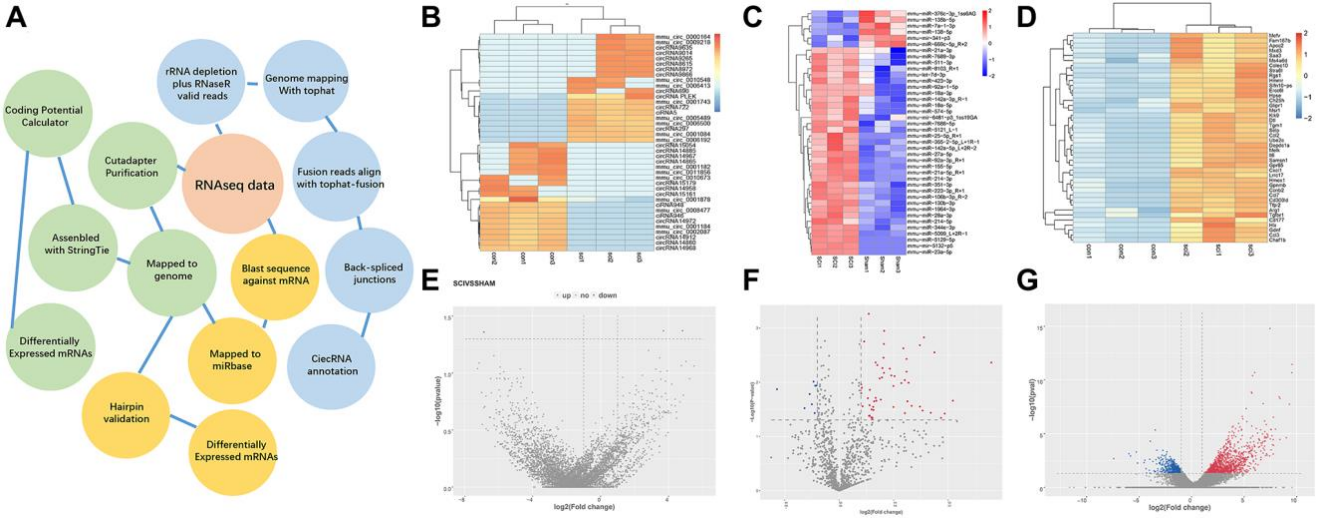
**Overview of differentially expressed ncRNAs and mRNAs.** (**A**) Overview of the analysis pipeline. (**B**–**D**) Hierarchical cluster analysis of significantly upregulated and downregulated ncRNAs and mRNAs. Each column represents a sample and each row represents an ncRNA or mRNA. Red represents high relative expression and blue represents low relative expression. (**E**–**G**) Volcano plots were used to visualize differential expression after SCI. The red and blue points represent upregulated and downregulated ncRNAs and mRNAs, respectively, with statistical significance. Significance was defined as *p* < 0.05.

A workflow of the bioinformatics analysis pipeline for the sequencing and screening of circRNAs, miRNAs, and mRNAs is shown (Figure 1A). The top 40 differentially expressed circRNAs (Figure 1B), miRNAs (Figure 1C), and mRNAs (Figure 1D) between the SCI group and the Sham group are summarized in a clustering map (*p* < 0.05) and volcano plot (Figure 1E–1G). Among the dysregulated loci, 31 circRNAs were upregulated and 29 circRNAs were downregulated, 93 miRNAs were upregulated and 57 miRNAs were downregulated, and 1417 mRNAs were upregulated and 265 mRNAs were downregulated in SCI samples compared to the Sham group.

### Validation of circPlek expression

**Figure 2:**
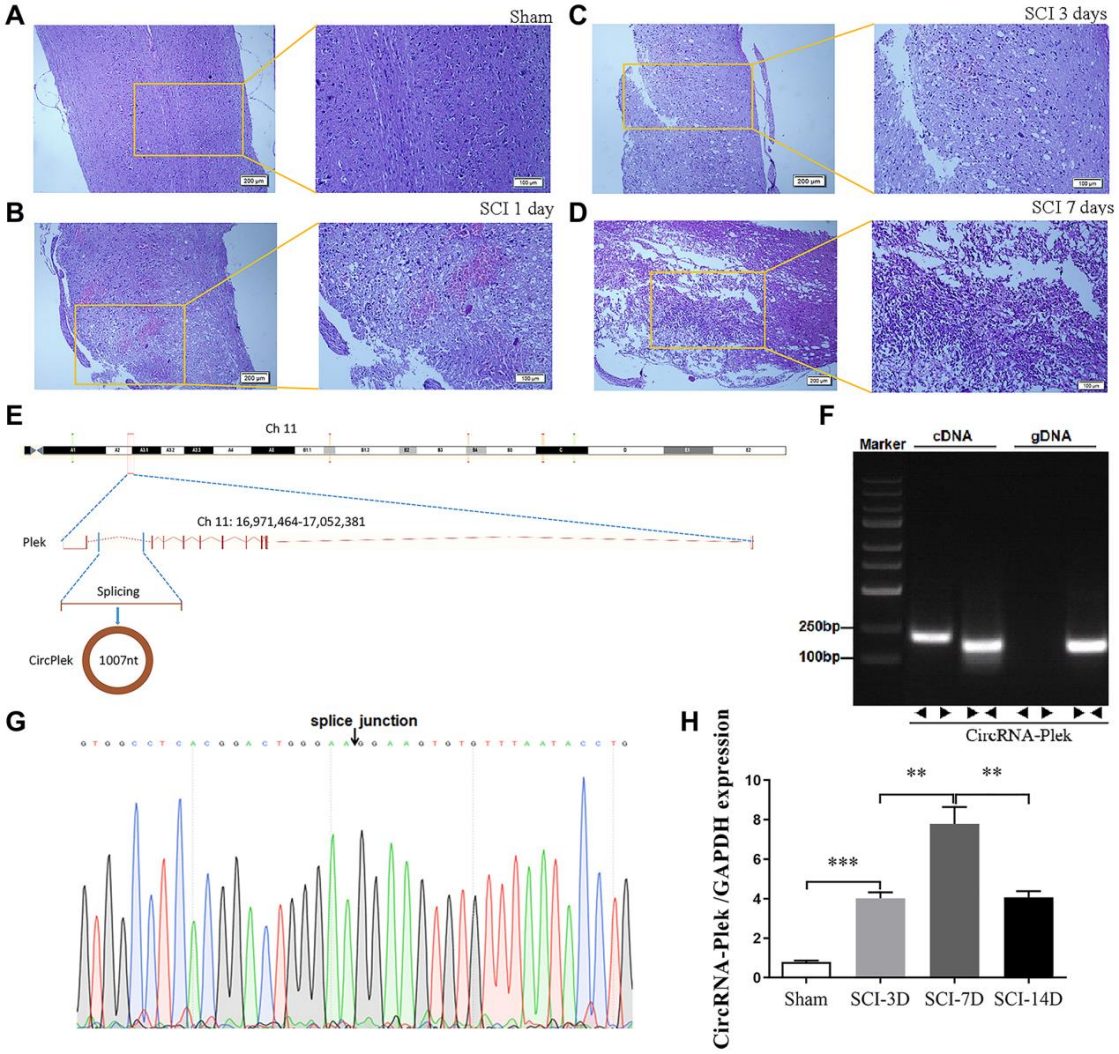
**CircPlek is upregulated in the spinal tissues after SCI.** (**A**–**D**) Animal models and sequencing processes, hematoxylin and eosin staining of spinal cord samples. Sham group (**A**), 1 day (**B**), 3 days (**C**), and 7 days (**D**) after spinal cord injury (SCI). (**E**) The schematic diagram indicates the genomic loci of the *Plek* gene and CircPlek. (Ch11:16971464–17052381). (**F**) CircPlek was validated in spinal tissues using RT-PCR. CircPlek could be amplified by convergent primers from both cDNA and gDNA. Divergent primers amplified CircPlek in cDNA but not gDNA. (**G**) The sequencing of PCR products obtained using divergent primers for CircPlek confirmed the head-to-tail junction point. (**H**) The relative expression of CircPlek was measured in spinal tissues on days 3, 7, and 14 post-SCI. Data are expressed as means ± standard deviations. ^**^p < 0.01, ^***^p < 0.001.

To verify the effect of SCI on ncRNA expression in the spinal cord lesion epicenter, a standard Allen’s drop model was constructed. The spinal tissues of inbred C57 mice were damaged using Allen’s impactor. Hematoxylin and eosin staining demonstrated that the blood–spinal cord barrier and the structural integrity of the spinal cord lesion epicenter were severely damaged by physical trauma, including rupture, hemorrhage, and inflammatory cell activation. The spinal cord structure of the sham group was intact without rupture and bleeding (Figure 2A). One day after the operation, there was visible rupture and bleeding at the site of injury (Figure 2B). Three days after the operation, the morphological damage to the tissue was not repaired, blood at the injury site was absorbed, and inflammatory cells gradually accumulated (Figure 2C). Seven days after the operation, a large number of inflammatory cells gradually gathered at the damaged area, cavitation was detected at the site of injury, and the shape and contour of the damaged tissue were repaired (Figure 2D). CircPlek is transcribed from the antisense strand of the *Plek* gene (Ch11:16971464–17052381) (Figure 2E). To further confirm the circularity of CircPlek, PCR results from cDNA and genomic DNA (gDNA) amplified using primers for CircPlek (divergent) and for linear transcripts (convergent) were compared. Only CircPlek was amplified by divergent primers in cDNA, with no amplification product using gDNA (Figure 2F). Sanger sequencing was performed to validate the sequence at the junction sites of CircPlek (Figure 2G). RT-PCR showed that the expression levels of CircPlek were significantly higher in the SCI group than in the Sham group within 14 days, and reached a peak at 7 days after injury (Figure 2H).

### Enrichment of biological functions and pathway networks

**Figure 3:**
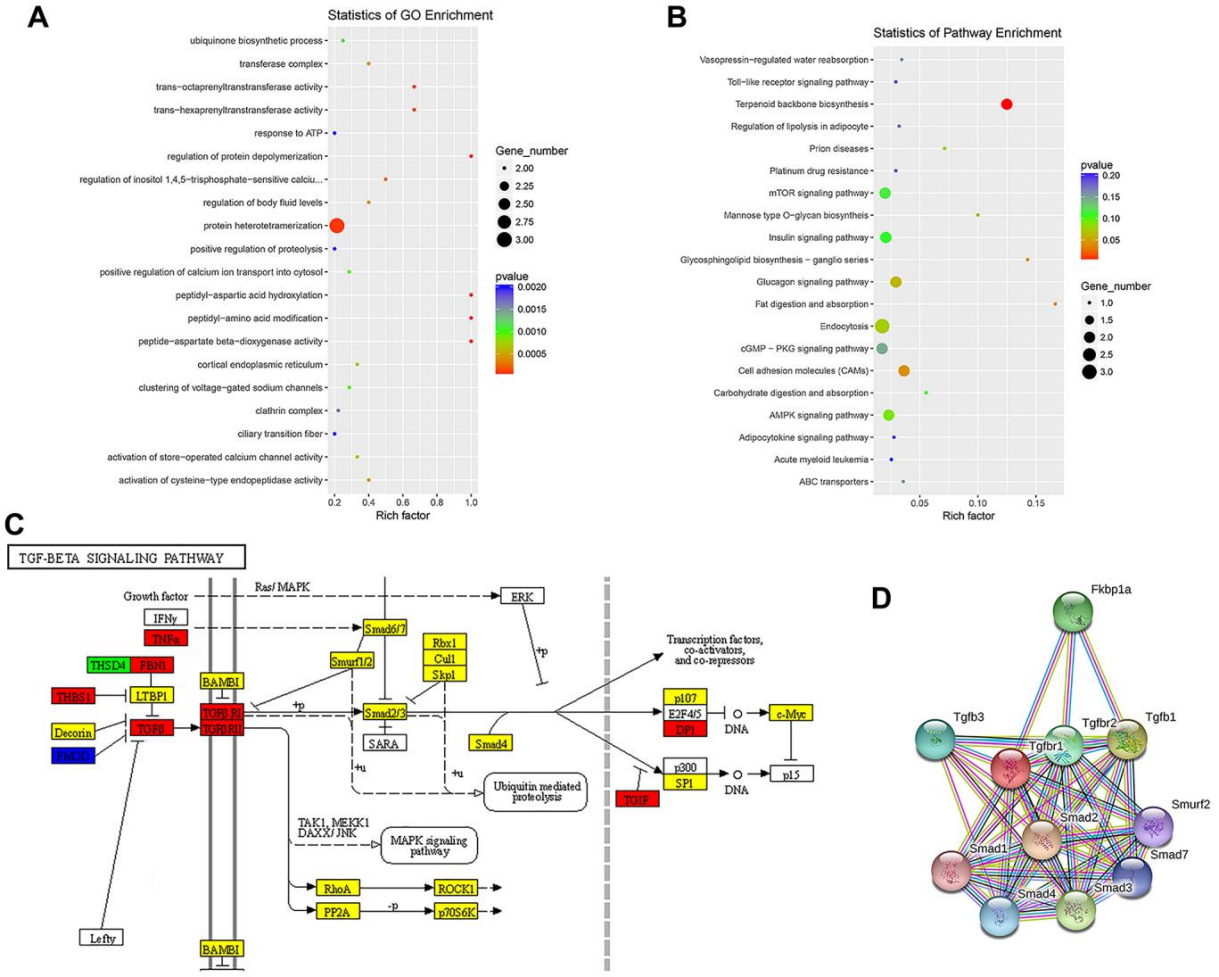
**Enrichment analysis of biological functions and pathways.** (**A**) Top 20 significantly enriched GO terms for differentially expressed ncRNAs are shown in a scatterplot. (**B**) Top 20 significantly enriched KEGG pathways for differentially expressed ncRNAs are shown in a scatterplot. (**C**) Mapping of the TGF-β/Smad pathway activated by SCI. In the map, genes encoding pathway proteins were significantly upregulated (red), upregulated without a significant difference (yellow), significantly downregulated (blue), or downregulated without a significant difference (green). (**D**) PPI analysis of TGF-β/Smad pathway-related proteins.

To evaluate the functional enrichment of differentially expressed circRNA targets after SCI, GO and KEGG pathway analyses were performed. The enrichment factor is the ratio of the observed number of differentially expressed genes to the expected number of genes; a higher enrichment factor indicates a greater degree of enrichment. A GO analysis of enrichment for biological processes, cellular components, and molecular functions showed that dysregulated circRNAs were associated with the regulation of protein depolymerization, peptidyl-aspartic acid hydroxylation, peptidyl-amino acid modification, peptide-aspartate beta-dioxygenase activity, trans-octaprenyltransferase activity, and trans-hexaprenyltransferase activity (Figure 3A). Moreover, significant KEGG pathways were associated with fat digestion and absorption, glycosphingolipid biosynthesis-ganglio-series, terpenoid backbone biosynthesis, mannose type O-glycan biosynthesis, prion diseases, and cell adhesion molecules (Figure 3B). The TGF-β/Smad pathway-related proteins THBS1, TNF-α, FBN1, TGF-β, TGF-βR1, TGF-βR2, DPI, and TGIF were significantly upregulated, whereas the expression of FMOD was significantly downregulated (Figure 3C). Since TGF-β, TGF-βR1, and TGF-βR2 are key factors in the activation of the pathway, we performed a PPI analysis of proteins in the TGF-β/Smad pathway. We detected complex regulatory relationships among pathway proteins. PPI networks were visualized using the STRING database (Figure 3D).

### Construction and confirmation of CircPlek/miR-135b-5p/TGF-βR1 interactions

**Figure 4:**
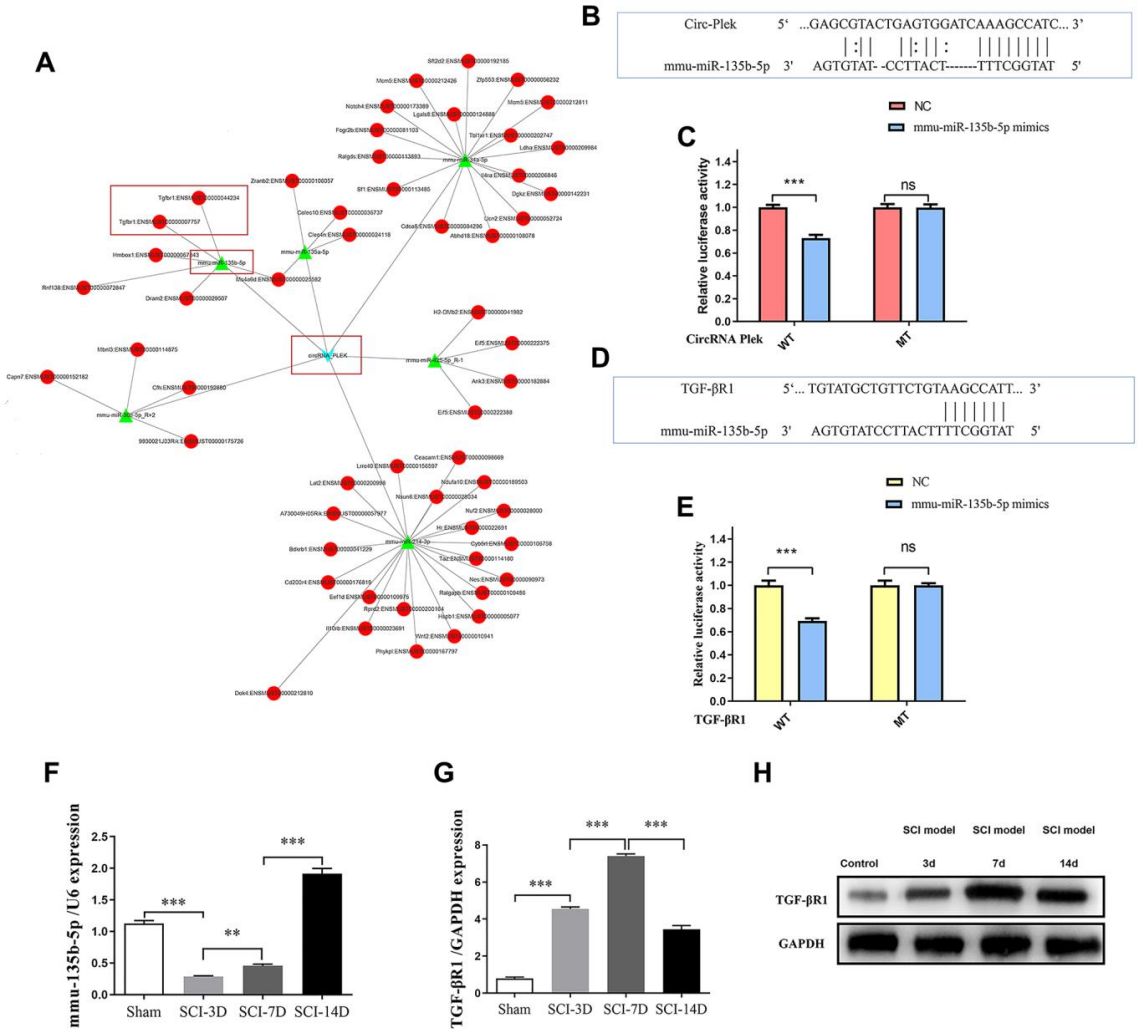
**CircRNA/miRNA/mRNA network analysis.** (**A**) The CircPlek/miR-135b-5p/TGF-βR1 axis was predicted (outlined in red). (**B**) An miR-135b-5p target site in CircPlek was predicted using TargetScan. (**C**) Relative luciferase expression of wild-type and mutant CircPlek bearing luciferase vectors co-transfected with miR-135b expression vectors. (**D**) A predicted miR-135b-5p target site in the 3′UTR of *TGF-βR1*. (**E**) Relative luciferase expression of the wild-type and mutant TGF-βR1 UTR-bearing luciferase vectors co-transfected with miR-135b-5p expression vectors. (**F**) The relative expression of miR-135b-5p was measured in spinal tissues using qRT-PCR on days 3, 7, and 14 post-SCI. (**G**) The relative expression of TGF-βR1 was measured in spinal tissues using qRT-PCR on days 3, 7, and 14 post-SCI. (**H**) The relative expression of TGF-βR1 was measured in spinal tissues using western blotting on days 3, 7, and 14 post-SCI. Data are expressed as means ± standard deviations. **p < 0.01, ***p < 0.001.

A panorama network was generated, consisting of eight dysregulated circRNAs, their target miRNAs, and target mRNAs of the miRNAs and a CircPlek/miR-135b-5p/TGF-βR1 axis was predicted (Figure 4A). The miR-135b-5p-specific target sites in the 3′UTR of TGF-βR1 and CircPlek were predicted using TargetScan and verified via a fluorescent reporter assay (Figure 4B–4E). The expression of miR-135b-5p was measured using qRT-PCR on days 3, 7, and 14 post-SCI. Compared with the control group, the expression of miR-135b-5p initially decreased and then increased; there was a sharp decrease in expression at 3 days after the operation and an increase at 7 days after the operation, but these levels were still lower than those in the Sham group. However, on day 14 day after the operation, the miRNA expression level was significantly higher than that of the Sham group (Figure 4F). Similarly, the relative expression of TGF-βR1 was measured using qRT-PCR. At 3 days after the operation, the expression of TGF-βR1 was significantly higher than that in the Sham group. The expression level reached its peak at 7 days after the operation and remained significantly higher than that in the control group, although it decreased at 14 days after the operation (Figure 4G). The expression of TGF-βR1 was also measured using western blotting and consistent results were obtained (Figure 4H).

### The CircPlek/miR-135b-5p/TGF-βR1 axis affects the activation of the TGF-β/Smad signaling pathway

**Figure 5:**
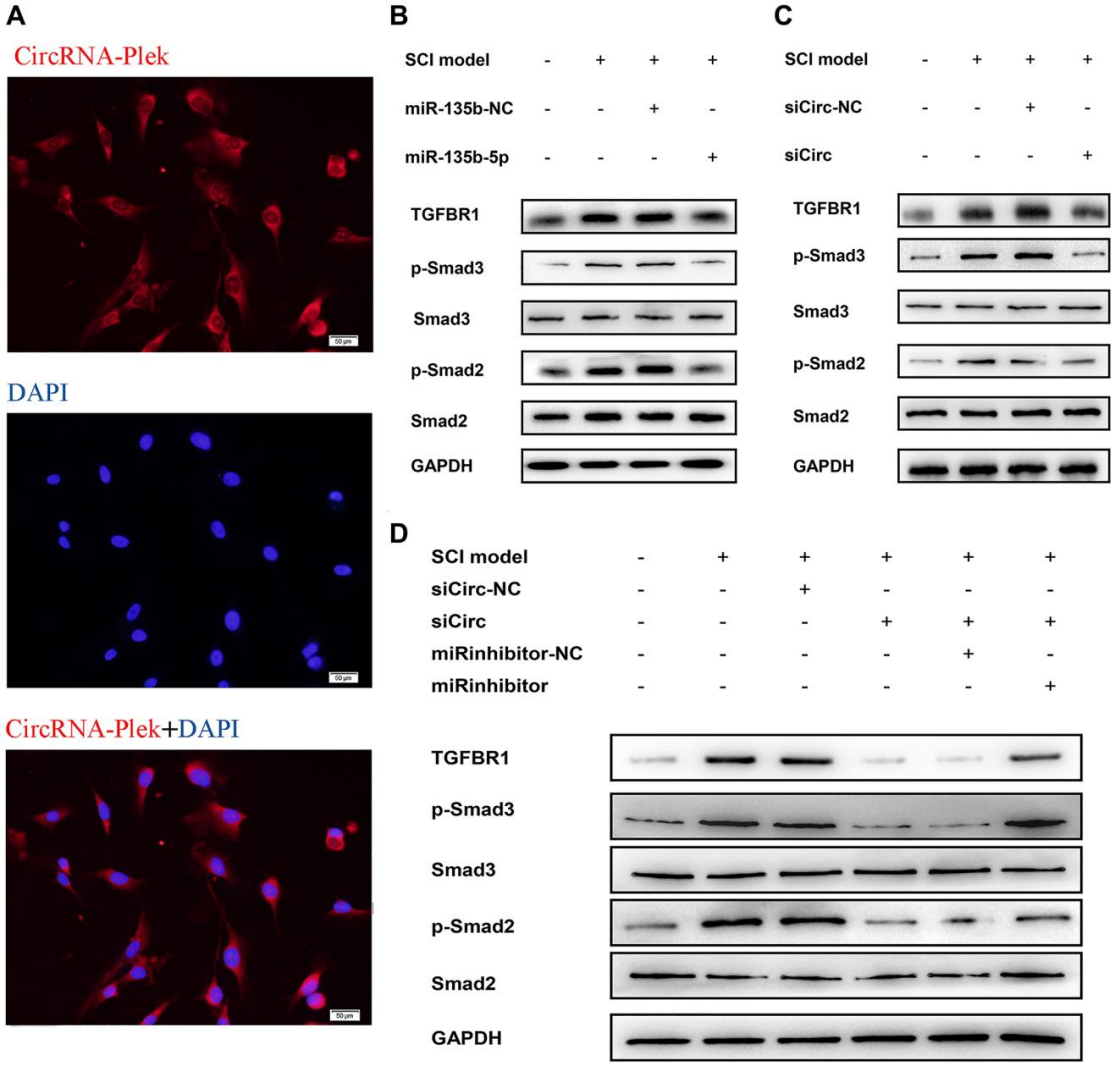
**CircPlek/miR-135b-5p/TGF-βR1 axis affects the activation of TGF-β/Smad signaling pathway.** (**A**) Fluorescence assays were performed to detect the location of CircPlek in spinal fibroblasts. Red represents CircPlek staining and blue represents nuclear DNA staining by DAPI. (**B**–**C**) Western blot analysis of the protein levels of Smad2, Smad3, p-Smad2, and p-Smad3 in spinal fibroblasts transfected with miR-135b-5p mimics (**B**) or siCircPlek (**C**). (**D**) After co-transfection of spinal fibroblasts with siCircPlek and an miR-135b-5p inhibitor with or without SCI, Smad2, Smad3, p-Smad2, and p-Smad3 protein levels were measured using western blotting.

To detect primary spinal fibroblasts, the expression of collagen I was measured by immunofluorescence staining (Supplementary Figure 1). A fluorescence *in situ* hybridization assay showed CircPlek localization in spinal fibroblasts (Figure 5A). The effects of miR-135b-5p on Smad signaling were analyzed in spinal fibroblasts treated with or without TGF-β1, and TGF-βR1 and the downstream proteins p-Smad2/3 and Smad2/3 were detected by western blotting. Treatment with miR-135b-5p mimics significantly inhibited the expression of TGF-βR1 and suppressed the phosphorylation levels of Smad2 and Smad3 (Figure 5B). siCircPlek had the same effect as miR-135b-5p mimics (Figure 5C). The co-transfection of spinal fibroblasts with siCircPlek and an miR-135b-5p inhibitor demonstrated that the miR-135b-5p inhibitor had an antagonistic effect on siCircPlek. The 135b-5p inhibitor had an antagonistic effect on circRNA expression, and the inhibitory effect of the circRNA on Smad signaling was offset to some extent after the use of the miR-135b-5p inhibitor (Figure 5D).

### The CircPlek/miR-135b-5p/TGF-βR1 axis regulates the progression of fibrosis

**Figure 6:**
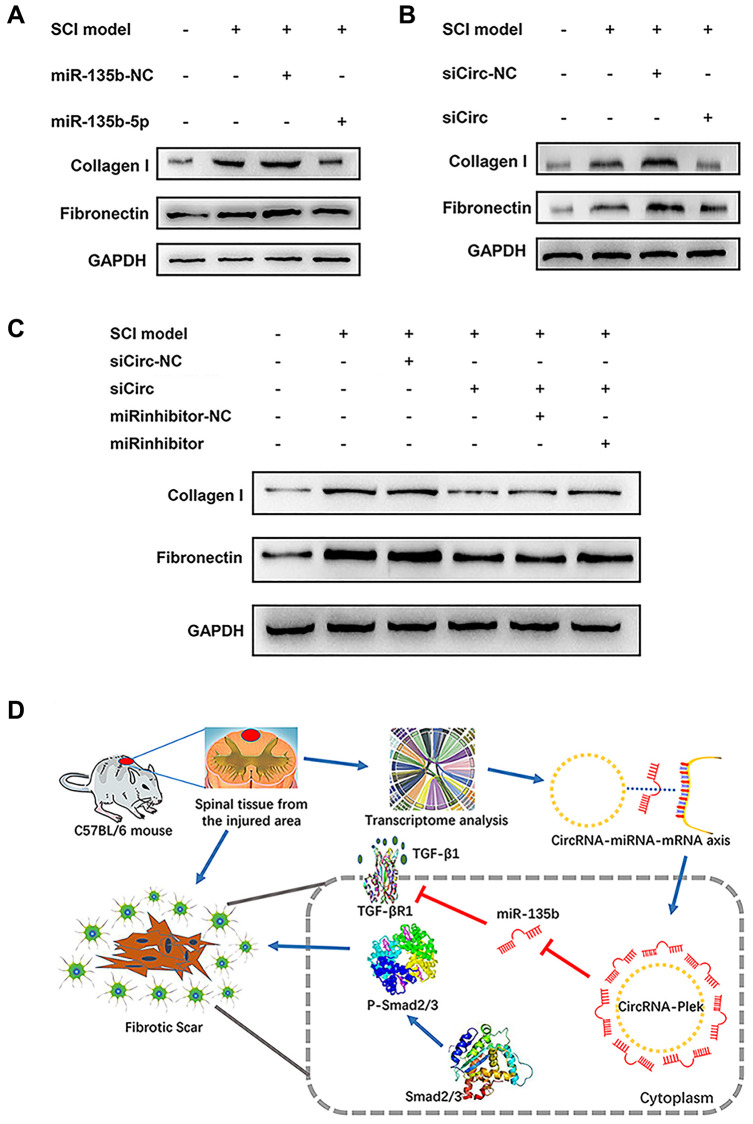
**CircPlek/miR-135b-5p/TGF-βR1 axis regulated the progression of fibrosis.** (**A**) Western blot showing fibronectin and collagen I protein levels in the control, SCI model, SCI + mimic NC, and SCI + miR-135b-5p mimics groups. (**B**) Western blotting showing fibronectin and collagen I protein levels in the control, SCI model, SCI + siCircRNA NC, and SCI + siCircPlek groups. (**C**) After co-transfection of spinal fibroblasts with siCircPlek and an miR-135b-5p inhibitor with or without SCI, fibronectin and collagen I protein levels were measured using western blotting. (**D**) Proposed model of the function of CircPlek in the regulation of fibrosis via the miR-135b-5p/TGF-βR1 axis in spinal fibroblasts. Under SCI, the expression of CircPlek was upregulated. Elevated CircPlek attracting miR-135b-5p, which bound to TGF-βR1, consequently inducing the activation of fibroblasts and fibrous scar formation.

The expression levels of the fibrosis-related proteins fibronectin and collagen I were measured using western blotting after transfection. miR-135b-5p mimics significantly suppressed fibrosis marker protein expression (Figure 6A), and siCircPlek had a similar effect (Figure 6B). After co-transfection with siCircPlek and the miR-135b-5p inhibitor, the antagonistic effect of the miR-135b-5p inhibitor against siCircPlek was observed (Figure 6C). After SCI, TGF-β overexpression stimulated its corresponding receptor TGF-βR1, thus activating the Smad pathway and aggravating fibrosis, leading to the formation of fibrous scars. The differential expression of miR-135b-5p after SCI can inhibit this process; however, miR-135b-5p itself is adsorbed and inhibited by CircPlek (Figure 6D).

## DISCUSSION

Inflammatory responses develop in the subacute phase of SCI [3]. Following exposure to the inflammatory environment post-injury, fibroblasts and astrocytes become activated and scars form [10]. Although the scarring process initially helps to resolve inflammation, scars become major impediments to axon regeneration [26]. Fibroblasts are the major connective tissue cells and provide a structural framework partially by the deposition of ECM components; they only invade or are produced in the CNS after injury and the progression of fibrosis with an increase in fibronectin, collagen, and laminin in the lesion epicenter [2, 3]. Recent studies have revealed specific roles of ncRNAs in many aspects of neuronal function and disease, such as neurite regrowth and neurodegeneration. Some ncRNAs directly influence the progression of fibrosis [27]. More generally, ncRNAs may contribute substantially to the pathogenesis of CNS injury based on their intrinsic properties, including the ability to regulate several related biological functions [28–30].

Although circRNAs were discovered more than 40 years ago, research on their function is still in its infancy [31, 32]. Unlike linear ncRNAs, circRNAs exhibit a unique enclosed loop structure. Owing to the lack of 5' and 3' ends, circRNAs are less sensitive to nucleases and are more stable than linear RNA. CircRNAs are expressed in various tissues in a wide range of taxa. In humans, circRNAs are derived from 14.4% of expressed genes and from about 1% of the total human RNAs [33]. The expression patterns of circRNAs are temporally, spatially, and tissue-specific. Most circRNAs are expressed at lower levels than those of their corresponding linear transcripts. However, their expression can be much higher than those of their corresponding linear transcripts in some cases, especially in the CNS. For example, circRNAs are highly expressed during nerve development, degeneration, transmitter release, and axon growth [34, 35]. In this study, GO and KEGG analyses of differentially expressed circRNA targets after SCI revealed that they are mainly involved in transcriptional regulation and protein phosphorylation, they are abundant in the cytoplasm, nuclei, exosomes, and plasma membranes, and play a role in protein, adenosine triphosphate, and metal ion binding. CircRNAs function by three general regulatory mechanisms. (1) CircRNAs affects the post-transcriptional regulation of miRNAs by acting as endogenous competing RNAs. (2) CircRNAs can bind to RNA-binding proteins, in turn affecting the level of gene expression. (3) CircRNAs contain an internal ribosome entry site for protein translation and expression [31]. It is currently believed that their role as a miRNA-adsorbing sponges to exert post-transcriptional regulatory functions is the most important function of circRNAs [17, 36, 37].

In this study, to further determine the roles of ncRNAs in the pathological process in SCI, the lesion epicenter of the T8–10 spinal cord was analyzed by a high-throughput sequencing approach to identify differentially expressed circRNAs, miRNAs, and mRNAs. miR-135b-5p showed the greatest decrease in expression after SCI. In a previous study, miR-135b-5p was defined as a proliferation, migration, hypertrophy, differentiation, and apoptosis-related miRNA [38–40]. Most importantly, miR-135b-5p has a neuroprotective function by targeting GSK3β3 and KLF4 [41, 42]. We predicted potential interactions between the significantly differentially expressed CircPlek, miR-135b-5p, and TGF-βR1. To our knowledge, CircPlek has not been detected previously. Accordingly, we first validated the circRNA by sequencing and PCR analyses. The expression levels of CircPlek, miR-135b-5p, and TGF-βR1 showed surprisingly consistent changes during a 14-day period after SCI. We therefore constructed the CircPlek/miR-135b-5p/TGF-βR1 interaction control axis and screened downstream signaling pathways. Based on KEGG and PPI analyses of related signaling pathways, we validated the roles of the Smad pathways. In our previous study, we explored vitro models of SCI using 10 ng/mL TGF to stimulate fibroblasts for 48 h [20]. In this study, we used the same model for *in vitro* experiments of the CircPlek/miR-135b-5p/TGF-βR1 axis. We confirmed that in TGF-β1-induced fibroblasts, the overexpression of miR-135b-5p or/and the inhibition of CircPlek inhibited the activation of fibrosis via the Smad pathway. Furthermore, inhibitors of miR-135b-5p had antagonistic effects on CircPlek.

Our study had some limitations. Biological regulatory pathways are highly complex. We verified the effects of proteins related to one pathway; however, other pathways, such as the PI3K/AKT signaling pathway [8], may also have regulatory effects. In addition, the regulation of ncRNA has a complex one-to-many or many-to-one relationship, this complex relationship is compounded by the complex cellular components of scar tissue, making the regulatory relationship even more complex. In this study, only one targeted interaction consisting of significantly differentially expressed genes was selected for verification, but the core circRNA might act on multiple mRNAs simultaneously with synergistic effects. Therefore, multi-cell, multi-target, and multi-pathway verification is a future research direction to elucidate the specific regulatory mechanism underlying spinal fibrosis.

In summary, by high-throughput sequencing and bioinformatics analyses, we identified a novel circRNA and constructed a related interaction network. It is reasonable to conclude that the CircPlek/miR-135b-5p/TGF-βR1 axis is involved in spinal fibrosis after SCI, and CircPlek may serve as a potential therapeutic target and prognostic marker. Our results support the key roles of interactions between ncRNAs and mRNAs in the pathophysiological process after SCI and provide a basis for the development of therapeutic strategies.

## Supplementary Materials

Supplementary Figure 1
