# Correction for: Circular RNA Plek promotes fibrogenic activation by regulating the miR-135b-5p/TGF-βR1 axis after spinal cord injury

**DOI:** 10.18632/aging.203271

**Published:** 2021-06-30

**Authors:** Wenzhao Wang, Dong He, Jianan Chen, Zhengdong Zhang, Shaoyi Wang, Yunpeng Jiang, Jianlu Wei

**Affiliations:** 1 Orthopedic Research Institute, Department of Orthopedics, West China Hospital, Sichuan University, Chengdu, Sichuan, China; 2 Department of Orthopedics, Qilu Hospital, Cheeloo College of Medicine, Shandong University, Jinan, Shandong, China; 3 Department of Neurology, Cheeloo College of Medicine, Shandong University, Jinan, Shandong, China; 4 Department of Orthopedics, Cheeloo College of Medicine, Jinan Central Hospital Affiliated to Shandong University, Jinan, Shandong, China; 5 Department of Orthopedics, The First Affiliated Hospital of Chengdu Medical College, Chengdu, Sichuan, China

**Keywords:** correction

Original article: Aging. 2021; 13:13211–13224.  . https://doi.org/10.18632/aging.203002

**This article has been corrected:** The authors requested to update affiliation 3 which was not completed. This correction does not change the content of the publication. The correct affiliation is provided below:

^3^Department of Neurosurgery, Shandong Provincial Hospital, Cheeloo College of Medicine, Shandong University, Jinan, Shandong, China

