# Supplementary material for: Circular RNA Plek promotes fibrogenic activation by regulating the miR-135b-5p/TGF-βR1 axis after spinal cord injury
Source: Aging (Albany NY). 2021 May 11;13(9):13211–24. doi: 10.18632/aging.203002 (PMC8148484; doi:10.18632/aging.203002)
Supplement: Supplementary Figure 1 [file aging-13-203002-s001.pdf]

## SUPPLEMENTARY FIGURE

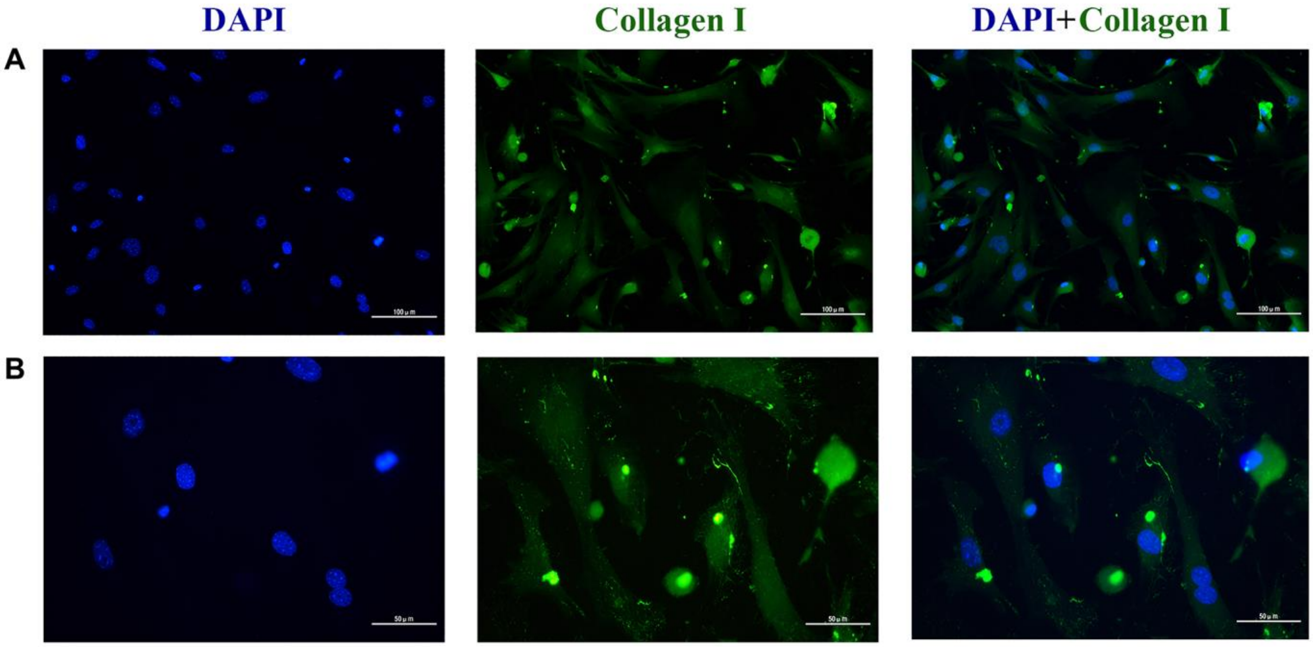


**Supplementary Figure 1. Identification of fibroblasts**. (**A**–**B**) Immunofluorescence staining of collagen I in spinal fibroblasts. Green represents collagen I staining, blue represents nuclear DNA staining by DAPI.
